# Monthly Summary

**Published:** 1881-01

**Authors:** 


### MONTHLY SUMMARY.

*Alloys and Solders.*—Alloys employed for joining metals together are termed "solders," and they are commonly divided into two classes; hard and soft solders. The former fuse only at a red heat, but soft solders fuse at comparatively low temperatures.

Of mixtures of metals which become liquid at temperatures at or below the boiling point of water, there are several known, some of which are placed in convenient order as follows :

1. *D'Arcet's* : Bismuth, 8; lead 5; tin 3 parts. This melts below 212° Fah.

2. *Walker's*: Bismuth 8; tin, 4; lead, 5 parts; antimony, 1 part. The metals should be repeatedly melted and poured into drops, until they can be well mixed previous to fusing them together.

3. *Onion's*; Lead, 3; tin, 2; bismuth, 5 parts. Melts at 187° Fah.

4. If to the latter, after removing it from the fire, one part of warm quicksilver be added, it will remain liquid at 170° Fah., and become firm and solid only at 140°

5. Another: Bismuth, 2; lead, 5; tin, 3 parts. Melts in boiling water.

Nos. 1, 2, 3, 5, are used to make toy spoons to surprise children by their melting in hot liquors. A little mercury (as in 4) may be added to lower their melting points.

Nos. 1 and 2 are especially adapted for making electrotype moulds. French cliche moulds are made with the alloy No. 2. These alloys are also used to form pencils for writing. Also as metal baths in the laboratory, or for soft soldering joints.

The following are the best receipts for the common solders:

*Hard spelter solder*: copper, 2 parts; zinc, 1 part. This solder is used for iron-work, gun-metals, etc.

*Hard silver solder*: silver, 4 parts; copper, 1 part; or silver, 2 parts; brass wire, 1 part. These are employed for fine work; the latter is most readily fusible.

For *brass-work*: equal parts of copper and zinc; or, for the finer kinds of work, silver, 1 part; copper, 8 parts; zinc, 8 parts.

For *steel*; silver, 19 parts; copper, 3 parts; zinc 1 part.

For *pewterers*: bismuth, 2 parts; lead, 4 parts; tin, 3 parts; or, bismuth, 1 part; lead 1 part; tin, 2 parts. The latter is best applied to the rougher kinds of work.

For *jewellers*: fine silver, 19 parts; brass, 10 parts; copper, 1 part; or, for joining gold: gold, 24 parts; silver, 2 parts; copper, 1 part.—*Druggists Circular.*

---

*The Tasting Power of the Tongue.*—The tasting power of the tongue is not regularly distributed over all parts of that organ. According to the unanimous judgment of physiologists, the back part of the tongue is best qualified for this function, while there is a difference of opinion as to the tip of the tongue. The old observers have repeatedly said that a tasting power in the tip is limited to certain persons, whereas more recent ones affirm its presence in all men. In experimenting on the so-called "reaction time," Herr Vintschgau lately met with a case of limited tasting power in the tongue-tip, and this led him to a thorough investigation of the subject. The observations were made with solutions of chloride of sodium, sugar, quinine, and citric acid. The results were as follows: There are persons who are capable of accurately distinguishing all principal tastes with the tip of the tongue alone; others perceive with certainty the qualities of sweetness, saltness, acidity, but less distinctly bitterness; others again can only with great difficulty distinguish tastes with the tip of the tongue; and finally there are individuals who cannot do this in the least.—*Drug. Circular.*

---

*Cervico-Bronchial Neuralgia Cured by Extraction of a Wisdom Tooth.*—On the 18th of February I was consulted by a patient who was suffering from severe neuralgic pains in the left temporal region of the head, and extended to the neck and arm on the same side. The patient described the pains in the neck and shoulder as being so severe as to interfere with the use of his arm.

Judging the pain in the temporal region to proceed from reflex irritation of the "auriculo-temporal nerve," caused by its connection with the "inferior dental," both belonging to the third division of the fifth pair of nerves, I determined to extract a decayed left lower wisdom tooth, and on doing so completely relieved all pains, The pulp of the tooth was in a state of chronic inflammation.

Now, although the implication of the cervical and brachial plexuses in neuralgia caused by carious teeth is a fact well known to dental surgeons, I have never been able to obtain a satisfactory reason how it is that these nerves, which are spinal, should be affected by irritation of the maxillary nerves, which are cranial.

While perusing my anatomical studies on the head and neck, I made it my business to endeavor to find out any communication which might exist, either normally or abnormally, between the maxillary nerves and the cervical and brachial plexuses, but could trace no connection at all, and to the repeated and persistent questions which I put to the demonstrator of anatomy on the subject, I could get no more satisfactory answer than, "We know the sympathy between these nerves to be a practical fact, but have no means of accounting for it."—*Musgrave, in British Journal of Dental Science.*

---

*Anæsthesia by Rapid Respiration.*—Dr. Benjamin Lee relates in the *Phila. Med. Times* several cases in which an analgesia effect was produced by rapid and forcible respiration. It is a method that has been employed successfully for some time by Dr. Bonwill, a dentist of Philadelphia. Dr. Lee's cases were those in which abscesses were opened or teeth extracted. Analgesia was not produced in every case, but it was brought about often enough to show that the effect is a genuine one. The respirations have to be kept up for a minute or more.

*Fetid Sweating of the Feet.*—A simple and effective method of dealing with that annoying infirmity is suggested by a correspondent of the *British Medical Journal.* He says :—

All that is necessary is to strap the affected portions of the sole of the foot as smoothly as possible with tolerably wide straps of ordinary adhesive plaster—either emplastrum saponis or emplastrum plumbi. Every part should be completely covered, and with two layers of plaster if the complaint be very bad. The plaster should be taken off and renewed in three or four days, and ouce again at the expiration of a week, when the skin will be found to be quite healthy, having its normal yellowish appearance, and will also be quite dry. The odor ceases from the first application, and the patient will walk away in comfort. One may with confidence predict, even in the most severe case, a perfect cure in the time mentioned.

There seems to be in some persons a tendency to relapse after an interval of some weeks; but on the slightest sign of reappearance of the disease, it is only necessary to cover the patch with a single strap of plaster, which will at once arrest its progress, remove the fetor, and speedily exert its curative influence.—*Med. and Surg. Reporter.*

---

*Keeping Rubber Plates Clean.*—Dr. N. W. Kingsley is reported as having said that one of the greatest disadvantages of rubber plates is "the impossibility, almost, in ordinary hands, of keeping rubber absolutely clean;" that out of a thousand sets that came into his hands after having been worn, but one was absolutely clean. He believes the absence of cleanliness to be the cause of the sore mouths we occasionally met with. Although the statement is rather broad, I believe it to be in the main correct, and I think I can point out the reason why: not one set in twenty is properly hardened in vulcanizing; consequently the polish wears off easily, and then to keep such a set clean is impossible. A set of teeth vulcanized at 320° for fifty-five minutes as recommended by the manufacturers is not fit to enter anybody's mouth. On the other hand, a set of teeth well hardened and highly polished can be kept clean as easily as a gold set, and far easier than celluloid.—*Guttman, in Dental Cosmos.*

---

*The Dangers of Chloroform.*—Two deaths following the administration of chloroform for medical purposes have recently occurred; one, that of a lady of Liverpool, took place in the surgery of a dentist, the deceased being described by her regular medical attendent as of "sound constitution." No blame can possibly be attached to any one, but the accident serves to point to the necessity for abandoning chloroform in favor of some anæsthetic not so often attended with fatalities.—*Medical Press and Circular.*

